# Deficiencia visual y neurológica posterior a la disfunción del sistema de derivación ventrículo-peritoneal: reporte de caso

**DOI:** 10.7705/biomedica.5657

**Published:** 2020-10-23

**Authors:** Valentina Duque, Laura Chaverra, Juanita Cury, María Carolina Portela, Juan Camilo Suárez-Escudero

**Affiliations:** 1 Escuela de Ciencias de la Salud, Facultad de Medicina, Universidad Pontificia Bolivariana, Medellín, Colombia; 2 Línea de Investigación en Discapacidad, Grupo de Investigación en Salud Pública, Escuela de Ciencias de la Salud, Facultad de Medicina, Universidad Pontificia Bolivariana, Medellín, Colombia

**Keywords:** parálisis cerebral, hemorragia cerebral, hidrocefalia, deficiencia visual, rehabilitación neurológica, derivación ventrículo-peritoneal, atrofia óptica, Cerebral palsy, cerebral hemorrhage, hydrocephalus, vision, low, neurological rehabilitation, ventricle-peritoneal shunt, optic atrophy

## Abstract

Neurological visual impairments in children have multiple causes, some of them reversible while others are not. Hydrocephalus is one of the most important and common ones as it can result in permanent impairment. There are multiple causes of hydrocephalus, intraventricular hemorrhage being the main one. This generally occurs when the germinal matrix bleeds and is very common in preterm newborns.

We present the clinical case of a patient with cerebral palsy, intraventricular hemorrhage, and hydrocephalus as a result of a preterm multiple pregnancy who developed optic atrophy during childhood secondary to ventricle-peritoneal shunt dysfunction. During the rehabilitation and treatment period, she received neurorehabilitation sessions, which improved her visual acuity and capacity. We found similarities and differences with other cases and we confirmed the importance of the treatment chosen for the recovery of visual capacity.

La retina y el nervio óptico se derivan del prosencéfalo y comparten su origen embriológico con el encéfalo. Las alteraciones congénitas del sistema nervioso central pueden estar asociadas con deficiencias visuales de origen neurológico. Además, dada la continuidad del espacio subaracnoideo y la vaina del nervio óptico, la vía visual anterior está sujeta a los cambios de la presión intracraneal, lo que resalta la importancia de tener en cuenta el sistema visual al revisar y hacer el seguimiento de condiciones intracraneales congénitas o adquiridas.

El sistema visual en los primeros siete años de vida se encuentra en proceso de maduración y es muy sensible a las lesiones, especialmente al aumento de la presión intracraneal, con posibles deficiencias permanentes. La elevación de la presión intracraneal aparece en situaciones en las que se produce daño cerebral por causas hipóxicas, metabólicas, tóxicas o traumáticas (1).

La incidencia estimada de la hidrocefalia es de 0,8 casos por cada 1.000 nacidos vivos en el mundo (2,3). Se considera un problema de salud pública, pues se estima que anualmente ocurren más de 380.000 nuevos casos. Además, el peso de la enfermedad en cuanto a discapacidad excede en gran medida la causada por tuberculosis, cardiopatía reumática y ceguera, entre otras (3). Las alteraciones visuales por hidrocefalia se deben mayoritariamente a la hipertensión intracraneal secundaria (4). A diferencia de los daños directos, los daños indirectos del nervio óptico –en el caso de la hidrocefalia– tienen más posibilidades de tratamiento y un mejor pronóstico (5).

Una de las principales causas de aumento de la presión intracraneal es la hidrocefalia, la cual se define como una distensión activa del sistema ventricular cerebral relacionada con el paso inadecuado del líquido cefalorraquídeo desde su punto de producción en el sistema ventricular hasta su punto de absorción y paso a la circulación sistémica (6). Se acompaña de aumento del tamaño ventricular en la mayoría de los casos y, si las fontanelas y suturas no están cerradas aún, también se incrementa el perímetro cefálico (2). Es una condición polimorfa tanto en su presentación clínica como en su etiología (2): cuando la hidrocefalia se debe a anomalías congénitas, frecuentemente se acompaña de trastornos oftalmológicos como la atrofia óptica, las ametropías, el estrabismo y el nistagmo, entre otros. La atrofia óptica es un hallazgo común en la hidrocefalia avanzada, con un aumento crónico de la presión intracraneal debido a la compresión del quiasma y del nervio óptico que puede resultar por dilatación del tercer ventrículo o aumento de dicha presión (4,7).

Entre las principales causas de hidrocefalia en recién nacidos, están la hemorragia intraventricular, los defectos del tubo neural, la estenosis del acueducto, la malformación de Chiari de tipo 2, los tumores cerebrales y el síndrome de Dandy-Walker.

La hemorragia intraventricular en infantes es la principal causa de hidrocefalia infantil (8) y, en recién nacidos prematuros, se debe al sangrado en la matriz germinal, una estructura en la pared ventricular subependimaria que genera células progenitoras neuronales para la corteza suprayacente e involuciona hacia la semana 32 de edad gestacional ajustada. Se trata de una estructura muy irrigada que presenta una rápida angiogénesis durante este periodo en los recién nacidos prematuros y, ante la inmadurez del sistema cardiovascular y del neurológico, hay un gran riesgo de que la matriz germinal presente sangrado con cambios hemodinámicos. La incidencia de la hemorragia intraventricular aumenta en relación inversamente proporcional al peso al nacer y la edad gestacional ajustada. En la mayoría de los casos, la hemorragia ocurre en las primeras 72 horas de vida, cuando los recién nacidos prematuros son bastante inestables clínica y hemodinámicamente (2).

Las distintas modalidades de tratamiento de la hidrocefalia pueden presentar fallas, especialmente mecánicas, y ello puede llevar a nuevos aumentos de la presión intracraneal que afectan la vía óptica. La vía óptica anterior puede lesionarse debido a la isquemia secundaria al aumento de la presión intracraneal o por una dilatación acentuada del tercer ventrículo que comprima el quiasma. Generalmente, en los niños que presentan disfunción de la derivación, los daños son transitorios más que permanentes, pero su recuperación visual puede tardar entre tres y cinco años. El daño irreversible se ocasiona con mayor frecuencia por la oclusión de las arterias cerebrales posteriores durante la herniación transtentorial, la cual produce el infarto de los lóbulos occipitales, pero, incluso en estos pacientes, es común cierto grado de recuperación visual (9). La detección temprana de la sintomatología visual, los controles prenatales, el seguimiento del tamaño de los ventrículos por neuroimágenes y el tratamiento oportuno de la hidrocefalia, contribuyen significativamente a la reducción de las deficiencias visuales (4).

Se reporta el caso de una paciente con parálisis cerebral infantil, hemorragia intraventricular e hidrocefalia, producto de un embarazo múltiple con prematuridad extrema, con deficiencia visual secundaria a disfunciones múltiples del sistema de derivación ventrículo-peritoneal.

## Presentación del caso

Se trata de una paciente de 12 años, tercera en nacer de un embarazo múltiple (cuatrillizas) de madre primeriza bajo tratamiento de fertilización asistida. El nacimiento fue por cesárea en la semana 26 debido a preeclampsia diagnosticada en la semana 24 de la edad gestacional ajustada. El peso al nacer fue de 880 g y estuvo hospitalizada durante 107 días con necesidad de respiración asistida por más de un mes.

Al nacimiento, presentó conducto arterial persistente, hipoxia y hemorragia intraventricular que condujo a ventriculomegalia con posterior hidrocefalia, sepsis y presión intracraneal espástica de predominio izquierdo. Se sometió a intervención quirúrgica debido al conducto arterial persistente y requirió un dispositivo de acceso ventricular para el manejo de la hidrocefalia con posterior derivación ventrículo-peritoneal a los tres meses de vida. Además, fue intervenida quirúrgicamente por estrabismo horizontal a los tres años y se le administró toxina botulínica de tipo A (50 U inicialmente) para la espasticidad producida por la presión intracraneal a partir de los cinco años en el miembro superior izquierdo. A los seis años, se inició la fisioterapia enfocada en el control motor de las cuatro extremidades y se entregó la órtesis pierna-pie, la cual continúa hasta el día de hoy. Además, se le hizo evaluación psicológica regularmente, ya que la paciente presentaba problemas de adaptación, socialización, impulsividad y reactividad. En la evolución se detectaron retraso global del desarrollo, problemas relacionados con la limitación de las actividades, trastorno articulatorio y desequilibrio muscular orofacial, pero no hubo episodios convulsivos.

**Figura 1:**
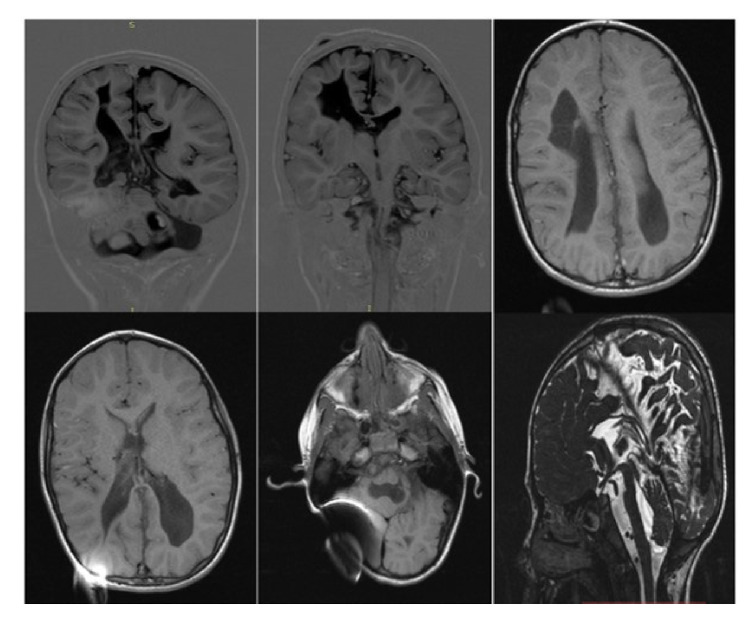
Resonancia magnética cerebral anterior a la disfunción del sistema de derivación ventrículo-peritoneal: cambios morfológicos con ventriculomegalia, adelgazamiento del cuerpo calloso y pérdida difusa del volumen de la sustancia blanca de ambos hemisferios cerebrales, predominantemente en el lóbulo frontal derecho y en ambos lóbulos occipitales, hallazgos asociados con extensas áreas de encefalomalacia en ambos hemisferios cerebelosos (mayor compromiso del lado izquierdo), así como una importante disminución del volumen del mesencéfalo, la protuberancia y los pedúnculos cerebelosos bilateralmente y estenosis del acueducto de Silvio

En cuanto al neurodesarrollo, tuvo sostén cefálico antes de los tres meses, sedestación y dominancia manual temprana a partir de los seis meses, control de esfínteres desde los tres años y marcha a los cuatro años. El examen neurológico a los siete años, anterior a la primera disfunción de la derivación ventrículo-peritoneal, se resumió así en la historia clínica: “Alerta, lenguaje coherente, dislalia y disartria, espasticidad Ashworth 1 en hemicuerpo derecho y Ashworth 2 en hemicuerpo izquierdo, perímetro cefálico de 48 cm, buen contacto y seguimiento visual, limitación para la extensión del pie izquierdo y leve hiperreflexia en miembro inferior ipsilateral”. La figura 1 corresponde a la imagen de resonancia magnética cerebral a esa edad.

**Figura 2:**
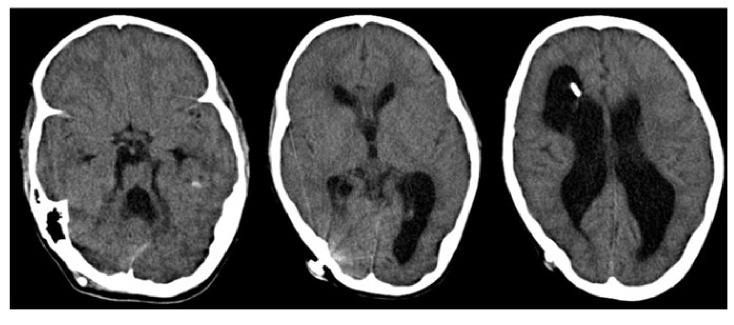
Tomografía computarizada que evidencia ventriculomegalia secundaria a cambios retráctiles del parénquima por encefalomalacia extensa con predominio frontal derecho y cerebeloso. No se observa edema transependimario ni obliteración del espacio subaracnoideo.

A partir de los diez años, la paciente experimentó tres episodios sugestivos de falla del sistema de derivación ventrículo-peritoneal. En el cuadro 1 se resume cada uno de ellos, señalando la edad de la paciente, la presentación clínica, los estudios radiológicos, la conducta, y las deficiencias neurológicas y visuales presentes (figura 2).

**Cuadro 1:** Aspectos de sospecha y falla confirmada del sistema de derivación ventrículo-peritoneal

**Aspecto**	**Edad**	**Clínica**	**Estudios radiológicos**	**Conducta**	**Deficiencia**
Primer episodio	10 años	Emesis, cefalea y síntomas visuales no especificados, motivo por el que se sospechó disfunción de la derivación ventrículo-peritoneal	TC cerebral: ventriculomegalia, encefalomalacia extensa con predominio frontal derecho y cerebeloso (figura 2) Imagen de resonancia magnética cerebral: pérdida difusa del volumen de la sustancia blanca de ambos hemisferios cerebrales, mínimo paso del LCR y ligera variabilidad sisto-diastólica de este	Revisión y cambio del sistema de derivación ventrículo-peritoneal	Paresia del VI nervio craneal izquierdo, reactividad pupilar lenta bilateral, disminución sustancial de la agudeza visual y de la capacidad de reconocer colores; se concluye que hay daño irreversible del nervio óptico.
Segundo episodio	11 años	Emesis, cefalea y síntomas visuales no especificados. En el examen físico se palpa derivación sin alteraciones, reservorio de derivación ventrículo-peritoneal depresible, pupilas simétricas con reacción lenta a la luz y agudeza visual sin percepción de luz y sin paresia del VI par	Radiografía de cráneo: se concluye que el sistema de la válvula está funcionando adecuadamente.	No se cambió el sistema y se dio egreso.	Ninguna
Tercer episodio	11 años (2 meses después segundo episodio)	Cefalea, emesis, nistagmos y alteración del estado de conciencia En el examen físico se palpan cámara y antecámaras de la válvula de derivación ventrículo-peritoneal colapsadas y sin llenado. Clínicamente, se considera que la paciente cursa con ventrículos rígidos y disfunción del sistema de derivación ventrículo-peritoneal.	TC de cráneo simple: catéter ventricular en adecuada posición, sin aumento de la ventriculomegalia, con cisuras	Revisión del sistema de derivación ventrículo-peritoneal confirma el estado disfuncional. Se inserta nuevo sistema de derivación con válvula programable de Hakim.	Persistencia del nistagmo, reflejo fotomotor directo lento bilateral, con mejor reacción en el ojo izquierdo

TC: tomografía computarizada; LCR: líquido cefalorraquídeo

Desde el primer episodio de falla del sistema de derivación ventrículo-peritoneal, se iniciaron las terapias de neurorrehabilitación sensorial visual con ejercicios de reconocimiento de color y figura, seguimiento y localización espacial y corporal, y se logró inicialmente que la paciente hiciera seguimiento con la mirada, lo que indicaba una mejoría de su condición, sin embargo, no logró evitar obstáculos al desplazarse. Se inició entonces el tratamiento con oclusión monocular y se logró un mayor reconocimiento con el ojo izquierdo. A pesar de los avances que presentó con la neurorrehabilitación visual, se sospechó daño irreversible del nervio óptico por hipertensión intracraneal.

Con las sesiones de neurorrehabilitación visual, se obtuvo cierta mejoría: la paciente aumentó parcialmente su capacidad de fijación visual, hizo movimientos de seguimiento de la luz con bajo contraste, comenzó a detectar objetos cercanos en movimiento y, en algunos momentos, siluetas y colores. Logró percibir luz y oscuridad, y a coordinar dedo y ojo con fuente de luz cercana. Conforme continuó con la rehabilitación, mejoró su agilidad para reconocer el movimiento de objetos cercanos y medianamente cercanos, reconoció los números a una distancia de 20 cm con movimiento horizontal y su agudeza visual le permitió contar sus dedos a 30 cm y discriminar entre el color rojo y el negro.

Posteriormente, en la consulta de optometría por deficiencia visual, se encontró que su visión excéntrica era mejor en el lado derecho y se le prescribieron lentes de visión cercana con las cuales logró identificar figuras, letras y números de tamaño grande.

Un mes después del segundo episodio, en el examen oftalmológico del fondo de ojo se encontró la mácula sana sin papiledema, discos ópticos semipálidos, y reflejos pupilares directo y consensual en ambos ojos pero con reactividad lenta. Además, se evidenció una mayor frecuencia de nistagmo, y la paciente reconocía el movimiento de las manos (vertical, horizontal y en círculo) y las siluetas. Su marcha era hemipléjica y sin deterioro en comparación con el patrón previo.

En el examen de oftalmología un mes después del tercer episodio, se registraron los siguientes signos: agudeza visual de lejos en el ojo derecho y percepción de la luz únicamente; con el ojo izquierdo percibía la luz y registraba visión cromática (utilizando la prueba de Ishihara); en el ojo derecho, solo había trazas del reflejo fotomotor directo. En ambos ojos, el diámetro pupilar en la luz era de 5 mm y de 6 mm en la penumbra, y los medios eran transparentes. En el fondo de ojo se observaron los discos ópticos redondos, con contornos definidos, pálidos, y excavación de 0,3 mm, pero no se observó cortocircuito óptico-ciliar. El sistema visual eferente y la motilidad ocular eran normales.

Un año después del tercer episodio, la niña acudió a consulta de neurooftalmología. En el examen se reportó gran ventriculomegalia sin edema transependimario.

En ambos ojos la agudeza visual le permitía percibir el movimiento de manos a 30 cm, pero la visión cromática era nula. El diámetro de las pupilas era de 5 mm en la luz y de 6 mm en la penumbra. El reflejo fotomotor directo era de 1 en el ojo derecho y de 2 en el izquierdo; se evidenciaba defecto pupilar aferente relativo en el ojo derecho. En el examen del fondo de ojo, los discos ópticos eran redondos, con contornos definidos, pálidos, excavación de 0,4 mm y no había cortocircuito óptico-ciliar. En el sistema visual eferente, los párpados y la motilidad ocular eran normales, y se producía nistagmo con la mirada extrema pendular.

**Figura 3:**
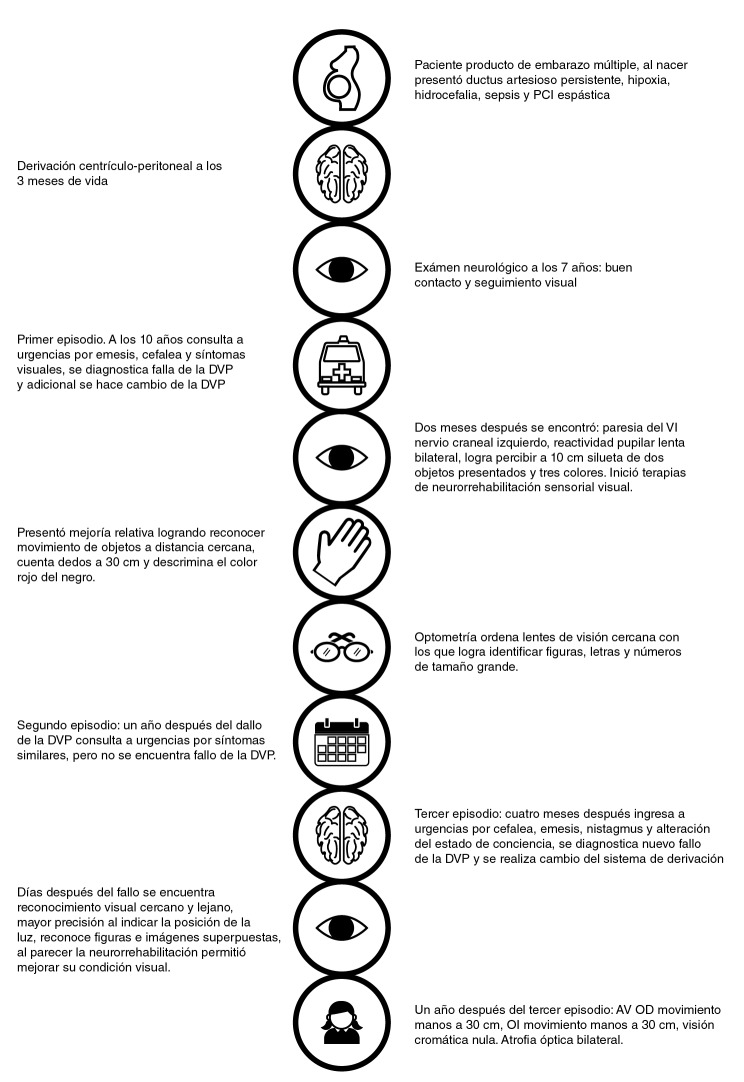
Evolución de la paciente

El diagnóstico final después del tercer episodio fue de atrofia óptica bilateral, parálisis cerebral mixta espástico-distónica de predominio izquierdo, hidrocefalia con derivación ventrículo-peritoneal, discapacidad intelectual y física (con deficiencia visual en seguimiento), leucomalacia periventricular y síndrome de ventrículos rígidos. En la figura 3 se presenta una síntesis gráfica de la evolución de la paciente, con los principales momentos clínicos y los resultados obtenidos.

## Discusión

La hemorragia intraventricular es la principal causa de hidrocefalia infantil y su manejo es crítico, ya que puede resultar en deficiencias permanentes (8). En los infantes prematuros, se registra entre el 64 y el 75% de la mortalidad por esta causa, entre el 42 y el 47% de las parálisis cerebrales infantiles, el 27% de los déficits intelectuales, el 37% de las discapacidades visuales y el 23% de las auditivas (8). Aproximadamente, un tercio de los infantes que sufren este tipo de hemorragia desarrollan dilatación ventricular y un tercio de ellos requiere derivación cerebroespinal permanente (8). La hemorragia puede limitarse a la pared ventricular (grado I), pero alrededor del 80% resulta en ruptura de la pared hacia el ventrículo (grados II, III y IV) (8). En este caso, la paciente presentó las dos condiciones en el contexto de una prematuridad extrema, lo que sugiere que la causa fue la presencia de la matriz germinal en las paredes ventriculares, que produjo aumento de la presión intracraneal e hidrocefalia. En estos casos, los infantes desarrollan hidrocefalia sintomática al cabo de varios días, con aumento gradual en su curso clínico, por lo que deben ser observados de cerca y se les debe medir el perímetro cefálico. Un aumento de 0,5 a 1 cm/día durante dos a tres días consecutivos, siempre sugiere hidrocefalia sintomática (8).

La derivación ventrículo-peritoneal es uno de los mecanismos más utilizados para tratar las hidrocefalias infantiles (9), pero, como cualquier dispositivo médico, puede generar complicaciones si su funcionamiento falla, ya sea por una obstrucción que impida el paso del líquido cefalorraquídeo hacia el peritoneo, porque el dispositivo se desplace o se desconecte, o por un fallo en el mecanismo (9).

Las manifestaciones clínicas de la paciente en el momento de la falla de la derivación ventrículo-peritoneal coinciden con los datos de un estudio descriptivo realizado en España en 1997, en el cual se encontró que los episodios eméticos y la cefalea constituyen dos de los tres síntomas más comunes después del fallo de la derivación (10), además de dolor ocular o alguna otra manifestación relacionada con la visión. Las manifestaciones clínicas oculares de esta paciente, como la disminución de la agudeza visual, fueron de curso subagudo, ya que empezaron a presentarse desde la primera falla de la derivación ventrículo-peritoneal hasta dos meses después del cambio de la válvula. En un caso similar en una joven de 15 años, el curso de aparición de los síntomas oculares fue de tres meses aproximadamente en los cuales la paciente refirió disminución de la agudeza visual en ambos ojos (11). Aparentemente, el tiempo de desarrollo de la disminución de la agudeza visual después de una falla de la derivación ventrículo-peritoneal fluctúa de semanas a meses (11).

La compresión de algún par craneal puede ser producto del aumento de la presión intracraneal que, en este caso, fue causada por la falla de la derivación ventrículo-peritoneal. El nervio abducente es el más comúnmente afectado en niños, ya sea por traumatismos, infecciones, neoplasias y, en general, por cualquier enfermedad que genere un incremento de la presión intracraneal (12). En el caso descrito se encontró que la paciente presentó una paresia del nervio abducente izquierdo que fue diagnosticada dos meses después del primer episodio de falla y del cambio de la derivación ventrículo-peritoneal. En la consulta realizada 11 meses después del diagnóstico de la parálisis del VI par craneal, aproximadamente, se encontró que ya estaba normal, es decir, hubo mejoría de la parálisis. En un caso en Estados Unidos una joven de 15 años acudió a urgencias por presentar diplopía de dos días de evolución; en el examen físico se encontró una parálisis del nervio abducente y gracias a varios estudios se determinó que la causa era una falla en la derivación ventrículo-peritoneal. La paciente recuperó la funcionalidad del nervio al día siguiente del cambio de su derivación ventrículo-peritoneal (12). En otro paciente reportado en un estudio retrospectivo publicado en el 2003, hubo parálisis bilateral del sexto par que posteriormente mejoró, pero el informe no especifica en cuánto tiempo se logró dicha mejoría (7).

Como se constató en este caso, el daño puede revertirse con el paso del tiempo y con las intervenciones adecuadas, pues la paciente empezó a presentar mejoría parcial de su capacidad visual semanas después de la primera falla de la derivación ventrículo-peritoneal. La paciente requirió terapias de neurorrehabilitación para fortalecer poco a poco las habilidades visuales y motoras que se habían deteriorado. El avance fue significativo y permitió que conservara algunas funciones visuales como la visión cromática, la sensibilidad al contraste y la agudeza visual cercana.

Se han reportado pacientes con problemas en la visión tales como las hemianopsias debidas a fallas en la válvula de la derivación ventrículo-peritoneal, que recuperaron la visión luego de ser intervenidos quirúrgicamente para reemplazar la válvula. En algunos casos, la mejoría se presentó a los pocos días de la intervención y hubo, incluso, resolución total del cuadro clínico (4,5).

En una serie de casos en Estados Unidos publicada en el 2003, se describe un caso similar al que se presenta: una paciente de 14 años cuya derivación ventrículo-peritoneal falló y dejó de percibir la luz a raíz de ello, pero al cabo de algunos meses recuperó en gran medida su agudeza visual (20/25 en el ojo derecho y 20/30 en el izquierdo) (7). Sin embargo, no es claro si su recuperación se debió a intervenciones terapéuticas o fue espontánea.

En cuanto a la duración de los síntomas visuales después de una falla de la derivación ventrículo-peritoneal, los reportes señalan que puede tardar entre 48 horas y un mes (7), dato que contrasta con el caso de la presente paciente, cuya recuperación tardó meses, al cabo de los cuales siguió presentando una deficiencia visual grave. En otro estudio de personas con discapacidad visual, se reportó que el 1,8% de los niños quedaron ciegos de manera permanente después de un episodio de elevación de la presión intracraneal por mal funcionamiento de la derivación (13).

En otro caso, de una paciente de 25 años nacida con hidrocefalia a quien se le colocó una derivación ventrículo-peritoneal a los siete meses de edad, ambos discos ópticos presentaron atrofia moderada (13). La paciente tuvo los mismos síntomas que la del presente caso (cefalea y náuseas) durante dos semanas y deterioro de la agudeza visual (con el ojo derecho en abducción solo reconocía movimiento de manos, y en el ojo izquierdo había aumento de la presión intraocular de 25 mm Hg y agudeza de 0,02).

En la tomografía computarizada (TC) también se evidenció dilatación ventricular, aunque no se registró aumento de la presión intracraneal con la punción de la válvula, la cual se hizo porque presentaba una obstrucción intermitente. Los síntomas no mejoraron y su agudeza visual empeoró, así que regresó ocho días después con ambas pupilas dilatadas, ausencia de reflejos a la luz directa y detección de estímulos de color verde únicamente. En la TC se hallaron los ventrículos aún más dilatados y, en esta ocasión, la presión intracraneal fue de 47 mm Hg al puncionar la válvula. Se removieron 30 ml de líquido cefalorraquídeo y con ello la paciente logró detectar de nuevo la luz, pero de forma leve. Se encontró una obstrucción en el catéter de la válvula, por lo que se cambió por un sistema de derivación con válvula programable de Hakim. Al día siguiente recuperó el reflejo directo frente a la luz y la percepción de estímulos oscuros; los síntomas sugestivos de presión intracraneal elevada mejoraron y, progresivamente, también su agudeza visual. En este caso, la ceguera se atribuyó a un aumento agudo de la presión intracraneal por obstrucción permanente del catéter. En la discusión de dicho artículo, se concluyó que la TC no es el método de diagnóstico adecuado para determinar el mal funcionamiento de la válvula, es preferible una angiografía con fluoresceína para determinar la perfusión de la retina (13). El aumento de la presión intracraneal comprime las arterias que irrigan el nervio óptico, lo que lleva a estasis circulatoria e isquemia. La ventriculomegalia también puede comprimir el infundíbulo, presionar el ángulo posterior del quiasma y causar ceguera (13).

Además de los problemas derivados de una falla de la derivación ventrículo-peritoneal, en niños con hidrocefalia es frecuente encontrar una eficiencia reducida para realizar tareas visuales y espaciales complejas, lo que se explicaría por una disfunción de las áreas posteriores del cerebro. En un estudio que incluyó 52 niños con hidrocefalia y 192 controles, se presentaron dificultades para reconocer formas (agnosia) en 23% del grupo afectado, para la percepción simultánea en el 21%, para percibir movimiento en el 14%, para reconocer colores en el 14%, así como para la orientación topográfica y el reconocimiento de rostros en el 6% (2).

Debe tenerse en cuenta que, en el presente caso, la paciente tenía una lesión cerebral desde el momento de su nacimiento debido a su prematuridad, antes del deterioro visual secundario a la falla de la derivación ventrículo-peritoneal. Su agudeza visual y otros aspectos de la vista se vieron afectados cuando presentó el segundo y el tercer episodios de falla del sistema de derivación, lo que produjo una disminución grave de la visión.

En un artículo publicado en 1991, se reportaron tres casos de infantes que presentaban hidrocefalia, daño de la derivación ventrículo-peritoneal y alteraciones visuales. Uno de los casos es el de una paciente nacida luego de un embarazo normal de 39 semanas, con mielomeningocele toracolumbar. En la TC se evidenció una malformación de Chiari II con dilatación ventricular acentuada; se le corrigió el defecto espinal y se le insertó una derivación ventrículo-peritoneal en el periodo neonatal. Desde el nacimiento se notó que era invidente, aunque estructuralmente no se encontraron anomalías oculares. Presentó disfunción de la DVP a los dos meses y medio, y a los cuatro meses; en ambas ocasiones, su visión mejoró rápidamente con la corrección. Dos meses después, su visión era considerada normal por sus cuidadores.

El segundo caso fue el de un paciente nacido a las 36 semanas de gestación luego de un embarazo normal. Se diagnosticó hidrocefalia poco después del nacimiento, se le hizo cefalocentesis y se colocó una derivación ventrículo-peritoneal en el periodo neonatal. La TC evidenció dilatación ventricular, estenosis del acueducto con anormalidad del cuerpo calloso y defectos migratorios de las cisuras de Silvio. Aunque estructuralmente no se encontraron anomalías oculares, se consideró ciego desde el nacimiento. A los cinco meses de edad aumentó su presión intracraneal, se reparó su derivación ventrículo-peritoneal y, al cabo de 48 horas, su visión mejoró hasta el punto de poder seguir objetos, pero continuó inatento visualmente. A los tres años, su agudeza visual era de 20/50.

El tercer paciente nació a término, pero requirió maniobras de reanimación en el momento del nacimiento. Se le diagnosticó un mielomeningocele lumbar alto, paraplejia e hidrocefalia que requirió derivación ventrículo-peritoneal. Aunque el examen ocular anatómico era normal, se le diagnosticó ceguera desde el nacimiento. A los siete meses se corrigió su estenosis sagital progresiva y su visión comenzó a mejorar en los primeros tres días a partir del procedimiento. A los seis años perdió la visión gradualmente a lo largo de cuatro a cinco meses, debido a la presencia intracraneal de un pequeño volumen de líquido, aunque su derivación ventrículo-peritoneal estaba funcionando. Luego de la corrección, su vista volvió a mejorar, con una agudeza de 20/40 en el ojo izquierdo y de 20/70 en el derecho. A los ocho años presentó disfunción de la derivación y empeoró su condición visual; hecha la corrección, su agudeza visual quedó en 20/40 en el ojo izquierdo y en 20/100 en el derecho (14).

En estos tres casos, la posible causa del deterioro visual fue el daño de la corteza visual y la radiación óptica (haz genículo calcarino). La rápida mejoría visual reportada sugiere que la fisiopatología de la ceguera pudo deberse a hipoperfusión prolongada de los lóbulos parietales y occipitales por incremento crónico de la presión intracraneal. Cuando esta se corrige, la perfusión a los lóbulos se restablece y las habilidades visuales se recuperan rápidamente (14), lo que podría explicar el presente caso ya que al corregirse la presión intracraneal, la paciente logró recuperar en cierto grado sus habilidades visuales.

Aún no hay un consenso sobre la definición del síndrome de ventrículo rígido. Se ha planteado que se trata de una condición caracterizada por cefalea durante 10 a 90 minutos, ventrículos pequeños en las imágenes diagnósticas y recambio lento del mecanismo de bombeo valvular (15). Puede considerarse que cualquier persona con derivación que presente cefalea intensa e imágenes que evidencien ventrículos normales o pequeños, puede tener el síndrome de ventrículos rígidos (15). La forma más grave de esta condición se denomina hidrocefalia de volumen normal, la cual se presenta con una presión intracraneal muy alta sin dilatación de los ventrículos en el momento de la falla de la derivación (16).

En cuanto a la fisiopatología, algunos autores proponen que la gliosis subependimaria contribuye a que se presente el síndrome porque esta endurece el cerebro. El hecho de que esta condición no aparezca antes de los dos años sugiere que los cambios asociados con la maduración, como la mielinización, la proliferación glial y la sinaptogénesis, contribuyen al desarrollo de las propiedades mecánicas del cerebro, y ello hace que el cerebro se torne más resistente a la dilatación ventricular. Por otro lado, la congestión venosa puede propiciar el síndrome. Otros autores sugieren, además, que un volumen ventricular excesivamente pequeño, combinado con una resistencia alterada a la salida del líquido cefalorraquídeo, puede impedir que el cerebro se acomode a las fluctuaciones normales del volumen vascular y precipitar aumentos más graves de la presión intracraneal (16).

La incidencia de los cambios en la visión asociada con fallas en la derivación es baja (menos del 2%) y el contraste entre los síntomas y los hallazgos clínicos puede generar confusión (13). La tríada conformada por la estenosis idiopática del acueducto, la derivación de larga data sin revisión y los ventrículos rígidos, aumenta el riesgo de presentar cambios en la agudeza visual asociados con la falla de la derivación (13). Es importante mencionar este síndrome, pues la presente paciente sufrió cambios en la agudeza visual después de cada episodio de falla de la derivación ventrículo-peritoneal, lo que se asociaría con las imágenes sugestivas de ventrículos rígidos.

La pérdida de visión asociada con la falla de la derivación resulta en una mayor mortalidad en pacientes con derivación ventrículo-peritoneal por hidrocefalia congénita. Cuando no sobreviene la muerte, el rápido deterioro puede causar deficiencias irreversibles y graves si no se detecta a tiempo. Por esta razón, se debe sospechar falla del funcionamiento de las válvulas de la derivación ventrículo-peritoneal en pacientes tratados por hidrocefalia que llegan a urgencias relatando síntomas como deterioro visual, cefalea y náuseas, incluso, si la derivación en la inspección clínica es depresible y aparentemente normal. El tratamiento oportuno de la elevación de la presión intracraneal puede evitar deficiencias irreversibles en la visión y, por lo tanto, mejorar la calidad de vida de los pacientes (13).

La visual es una de las categorías de discapacidad con mayor prevalencia mundial (cerca de 36 millones de personas con ceguera y 217 millones de personas con deficiencia visual) (17) y es de gran impacto por sus consecuencias en el funcionamiento y la calidad de vida. Cabe mencionar que muchas de las enfermedades que causan daño en la vía visual en la infancia son prevenibles, y lo más importante para el personal médico es el reconocimiento oportuno y la prevención del daño. Sin embargo, se presentan situaciones como la descrita en este reporte, en las cuales los esfuerzos deben orientarse a la rehabilitación, es decir, medidas de prevención terciaria en salud.

En conclusión, se presenta una paciente con deterioro visual significativo después de una falla de la derivación ventrículo-peritoneal, cuya evolución fue tórpida y atípica, posiblemente por los múltiples factores y variables inherentes a la paciente, como las condiciones neurológicas preexistentes que pudieron contribuir a su poca plasticidad cerebral, además de tres episodios de falla de su sistema de derivación. Aunque los síntomas de la paciente y su evolución inicial después de la primera falla son comparables con otros reportes de caso, la deficiencia persistió a pesar de la mejoría de los parámetros visuales, lo que es atípico comparado con la evolución de otros pacientes cuyos síntomas visuales, en su mayoría, mejoraron en días o meses. Quedaría por explorar la razón por la que la paciente en cuestión tardó meses en lograr la reducida mejoría visual que se obtuvo, y en qué medida influyeron las terapias de neurorrehabilitación comparadas con la mejoría espontánea presente en otros casos.
